# A Review of Toxicity Mechanism Studies of Electronic Cigarettes on Respiratory System

**DOI:** 10.3390/ijms23095030

**Published:** 2022-05-01

**Authors:** Lilan Wang, Yao Wang, Jianwen Chen, Peiqing Liu, Min Li

**Affiliations:** School of Pharmaceutical Sciences, Guangdong Provincial Key Laboratory of Chiral Molecule and Drug Discovery, National and Local Joint Engineering Laboratory of Druggability and New Drugs Evaluation, Guangdong Engineering Laboratory of Druggability and New Drugs Evaluation, Sun Yat-Sen University, Guangzhou 510006, China; wangllan3@mail2.sysu.edu.cn (L.W.); wangy666@mail2.sysu.edu.cn (Y.W.); chenjwen@mail.sysu.edu.cn (J.C.)

**Keywords:** conventional cigarette, electronic cigarette, mechanism study, respiration system, signal pathway

## Abstract

Electronic cigarettes (e-cigarettes) have attracted much attention as a new substitute for conventional cigarettes. E-cigarettes are first exposed to the respiratory system after inhalation, and studies on the toxicity mechanisms of e-cigarettes have been reported. Current research shows that e-cigarette exposure may have potentially harmful effects on cells, animals, and humans, while the safety evaluation of the long-term effects of e-cigarette use is still unknown. Similar but not identical to conventional cigarettes, the toxicity mechanisms of e-cigarettes are mainly manifested in oxidative stress, inflammatory responses, and DNA damage. This review will summarize the toxicity mechanisms and signal pathways of conventional cigarettes and e-cigarettes concerning the respiratory system, which could give researchers a better understanding and direction on the effects of e-cigarettes on our health.

## 1. Introduction

### 1.1. Brief Introduction to E-Cigarettes

Conventional cigarette seriously endangers human health, and the number of smokers is enormous. WHO reports that an estimated 1.3 billion people worldwide use tobacco products (https://www.who.int/news-room/fact-sheets/detail/tobacco (accessed on 26 July 2021)). Therefore, electronic cigarettes (e-cigarettes) have attracted attention as a new substitute for conventional cigarettes. E-cigarettes are often referred to as alternative nicotine delivery systems or electronic nicotine delivery systems. Unlike conventional cigarettes, the e-cigarette device is mainly composed of three parts, a battery, an e-liquid mixture cartridge, and an electronic heating atomizer to vaporize the e-liquid mixture to create an aerosol. The e-liquid typically contains propylene glycol (PG), vegetable glycerin (VG), nicotine, flavors, and other additives [1]. E-cigarettes came into the market in 2003 and were patented internationally in 2007. In the same year, e-cigarettes entered the US market and have gained popularity among cigarette smokers [2].

### 1.2. Evolution of E-Cigarettes

**Figure 1:**
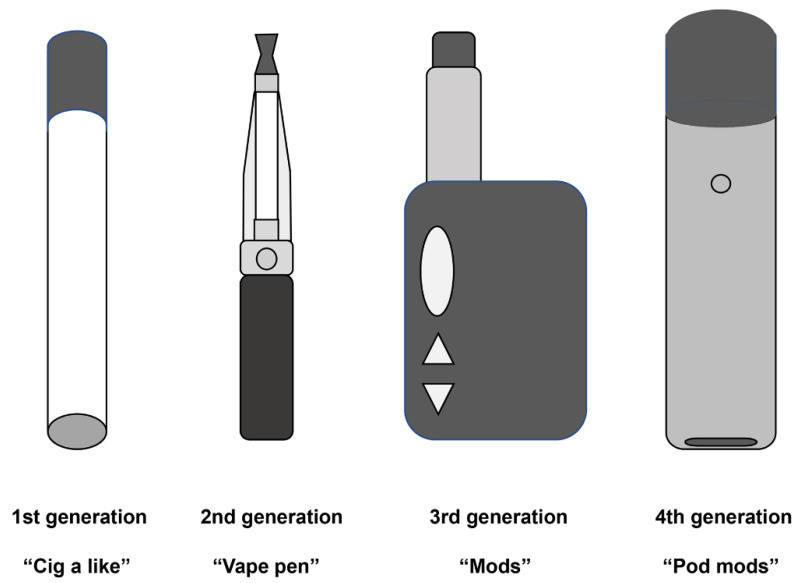
Schematics of the four generations of e-cigarettes.

As e-cigarette devices have evolved rapidly and new flavors have been introduced continuously, four generations of e-cigarettes can be identified in the market. The first generation is designed to mimic the appearance of combustible tobacco cigarettes, often called “cig-a-like”. The second generation has a much-larger-capacity battery and cartridge than the first generation, and its appearance is like a pen. The third generation shares no commonality with conventional cigarettes in appearance. It is available in several different sizes and shapes and has a customizable atomizer that allows users to adjust the atomization power. Therefore, the third generation is known as “Mods” [3]. The newest generation of e-cigarettes is a compact capsule-like device, also known as “Pod Mods”, attached via magnets that demand less energy and generate more steam. Pod Mods typically utilize nicotine salts rather than free-base nicotine used in the previous generations. Nicotine salts can cause high nicotine concentrations with few side effects compared with free-base nicotine [4]. The schematics of the four generations of e-cigarettes are shown in Figure 1.

### 1.3. Population Analysis of E-Cigarettes

Since the introduction of e-cigarettes in the United States in 2007, e-cigarettes have rapidly gained popularity among adults. Although the United States and the United Kingdom are the current major consumer countries of e-cigarettes [5,6], in China, as the largest tobacco producer and consumer worldwide, the use of e-cigarettes is growing rapidly [7]. Among Chinese adults aged 18 years and older, the estimated prevalence of the use of e-cigarettes increased from 1.3% in 2015 to 1.6% in 2019 in China [8]. Although smoking cessation is the most frequently reported reason in large-scale population analyses, the health benefits of e-cigarette use compared with smoking remain a focus of most other studies reported [9].

### 1.4. Composition Comparison of E-Cigarette and Conventional Cigarette Smoke

The chemical profiles of e-cigarette smoke are almost entirely different from those of conventional cigarette smoke, while nicotine is the main component found in both inhalants. E-cigarette aerosols generally contains fewer toxic components than conventional cigarette smoke [10]. Nicotine can be metabolized into the lung carcinogens nitrosamines. Nitrosamines and polycyclic aromatic hydrocarbons, two classes of known carcinogens, are the most critical toxic compounds in conventional cigarette smoke associated with many diseases, including cancer and pulmonary and cardiovascular diseases [11]. Toxic aldehydes and ketones produced as degradation products during e-cigarette liquid heating can adversely affect health [12]. E-cigarettes are a potential source of exposure to metals or metalloids [13]. Heavy metals and specific nitrosamines have been detected in the liquids and aerosols of e-cigarettes [14,15,16]. However, the levels of these components are generally much lower than in conventional cigarettes [17]. While the use of e-cigarettes reduces toxic components compared with traditional cigarettes, it remains a source of exposure to harmful substances. Differences in poisonous compounds in conventional cigarette and e-cigarette smoke are shown in Table 1.

### 1.5. Toxicity Comparison of E-Cigarettes and Conventional Cigarettes

Most of the current studies show that the toxicity of e-cigarettes is much lower than that of conventional cigarettes at the in vivo and in vitro levels [10,20,21,22]. Conventional cigarette smoking is associated with nearly every chronic inflammatory lung disease and is a causative factor in chronic obstructive pulmonary disease (COPD) [23]. However, E-cigarettes only recently entered the international market in 2007, and thus epidemiological data on the health effects of long-term vaping will take decades to acquire [24]. To determine the potential long-term harm of e-cigarettes, various methods and cell or animal models were utilized to assess the effects of e-cigarettes on cell function, production of inflammatory mediators, levels of oxidative stress, and DNA damage [21]. E-cigarettes are first exposed to the respiratory system after inhalation, and atomized e-cigarette smoke is not completely metabolized in the lung. Part of the inhaled substances are absorbed into the bloodstream and distributed throughout the cardiovascular, central nervous, and immune systems and so on. Studies to date have focused primarily on the respiratory system. Here, we review what is known thus far about the toxic mechanisms and the signal pathways of conventional cigarettes and e-cigarettes concerning the respiratory system.

## 2. Effects of Conventional Cigarettes on the Respiratory System

### 2.1. Conventional Cigarettes and Respiratory Diseases

Smoke particles from the burning of conventional cigarettes are the main harmful substances, which spread from the lungs into the blood and then into the body organs. Conventional cigarette smoking could cause adverse effects on human health and remains one of the leading causes of cancer-related deaths and respiratory diseases, including COPD, emphysema, bronchial asthma, and so forth (https://www.cdc.gov/tobacco/data_statistics/fact_sheets/health_effects/effects_cig_smoking/index.htm (accessed on 20 April 2017)).

Cigarette smoking can cause many types of cancer. About 90% of lung cancers in men and 80% of lung cancers in women are related to smoking, including squamous carcinoma, small cell lung cancer, adenocarcinoma, and large cell carcinoma, according to the World Cancer Report 2020 published by the World Health Organization (https://www.iarc.who.int/featured-news/new-world-cancer-report (accessed on 2 April 2020)). Smoking and aberrant epithelial responses are risk factors for lung cancer. In long-term cigarette smoke exposure, the infiltration of inflammatory cells to mucosa, submucosa, and glandular tissue is a major cause for the destruction of the matrix, blood supply shortage, and epithelial cell death. Conversely, cancer cells can regulate the proliferation of epithelial cells and generate new vascular networks [25].

COPD is one of the most common respiratory diseases, and its high prevalence makes it one of the leading causes of morbidity and mortality worldwide, which ranked third among the global age-standardized death rates [26]. Disturbed airflow with dyspnea, cough, and sputum production are the cardinal features of COPD [27]. Compared with nonsmokers, smokers have an accelerated lung function loss and a higher mortality rate. Additionally, smokers with COPD are more dependent on nicotine than normal smokers [28]. Furthermore, in addition to current smokers, children exposed to maternal smoking early are also at increased risk of developing COPD later in life [29].

Bronchial asthma is a chronic inflammatory airway disease characterized by airway hyper-responsiveness and reversible airway constriction, with a high incidence worldwide. Cigarette smoking can increase asthma morbidity, exacerbation severity, and mortality [30]. With severe asthma, smokers are associated with a higher rate of uncontrolled disease than nonsmokers [31]. Children exposed to long-term cigarette smoke have an increased risk of inducing asthma [32]. Cigarette smoke exposure during pregnancy is a significant factor in increasing the incidence of asthma, with a dose-dependent increase in asthma risk in offspring due to maternal smoking [33,34].

For smokers, cigarette smoking cessation can effectively reduce cigarette-related health risks. However, most smokers have difficulty quitting smoking due to their psychological and physical dependence on cigarettes. It is difficult for smoking cessation because smokers are dependent on nicotine, the primary addictive substance, and need to maintain a specific concentration of nicotine in the body to avoid nicotine withdrawal symptoms. Therefore, it is necessary to look for conventional cigarette substitutes or nicotine substitutes.

### 2.2. Conventional Cigarettes Related Toxicity Mechanisms and Signal Pathways

**Figure 2:**
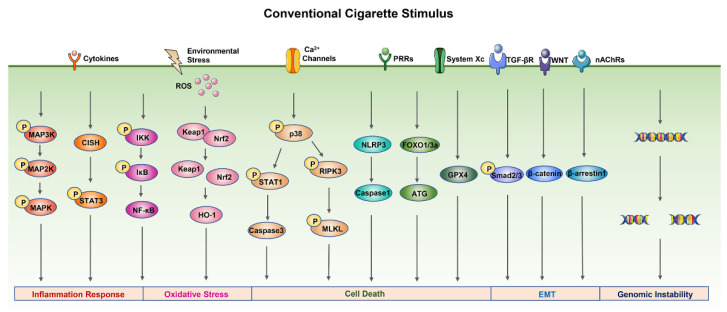
The main mechanisms of conventional cigarette smoke cytotoxicity. Cigarette smoke exposure can activate the MAPK, NF-κB, and STAT3 signal pathways to promote the release of inflammatory mediators. ROS and other oxidants caused by cigarette smoke can stimulate Nrf2 and NF-κB signal pathways to alter cellular redox homeostasis and lead to oxidative stress. Cell death caused by cigarette smoke exposure is related to the p38/STAT1/caspase-3 apoptosis signal pathway, p38/RIPK3/MLKL necroptosis signal pathway, NLRP3/caspase-1 pyroptosis signal pathway, GPX4 ferroptosis signal pathway, FOXO/ATG autophagy signal pathway, and so on. In addition, cigarette smoke exposure could promote ugh WNT/β-catenin, TGF-β/Smad2/3, or nAChR-dependent signal pathways and cause DNA damage. Abbreviation: ATG, autophagy-related proteins; CISH, cytokine-inducible Src homology 2-containing protein; FOXO, forkhead box class O; GPX4, glutathione peroxidase 4; HO-1, heme oxygenase-1; IκB, an inhibitor of NF-κB; IKK, IκB kinase; Keap1, Kelch-like ECH-associated protein 1; MAPK, mitogen-activated protein kinase; MLKL, mixed lineage kinase domain-like; nAChRs, nicotinic acetylcholine receptors; NF-κB, nuclear factor κB; NLRP3, nucleotide-binding domain-like receptor protein-3; Nrf2, nuclear factor erythroid-2-related factor-2; PRRs, pattern recognition receptors; RIPK3, receptor-interacting serine/threonine-protein kinase 3; ROS, reactive oxygen species; STAT, signal transduction and activator of transcription; TGF-βR, transforming growth factor-β receptors; WNT, wingless/integrase-1.

Conventional cigarette smoke is well known to be harmful to health. Several toxicity mechanisms have been implicated in cigarette smoke-induced damage, including inflammation response, oxidative stress, cell death, genomic instability, and epithelial–mesenchymal transition (EMT). Here, we offer a brief overview of the main mechanisms of conventional cigarette smoke cytotoxicity (Figure 2).

#### 2.2.1. Inflammation Response

Cigarette smoke exposure in vitro induces the release of chemokines and inflammatory mediators from human airway epithelial cells and neutrophils [22,35]. Additionally, cigarette smoke is involved in many cellular signaling pathways associated with inflammation response, including mitogen-activated protein kinases (MAPKs), nuclear factor kappa-B (NF-κB), and signal transducer and activator of transcription (STAT) [36,37,38].

MAPK signaling pathways are mainly involved in regulating inflammation response and the release of inflammatory cytokines in cells, including p38, ERK1/2, and JNK1/2. Cigarette smoke can significantly increase the ERK1/2, JNK1/2, and p38 phosphorylation and induce the release of the inflammatory cytokines IL-1β, IL-6, and IL-8 in a time-dependent manner [39]. It has been reported that the expression and activation of NF-κB are increased in COPD and associated with the increased release of NF-κB-dependent proinflammatory cytokines [40]. Cigarette smoke can activate the NF-κB signaling pathway by increasing the levels of phosphorylated IκB and phosphorylated IKK, initiating the inflammation response and, in turn, promoting the release of inflammatory mediators, such as IL-6, IL-8, and TNF-α [37]. STAT3 signaling is a significant pathway for cancer inflammation as it is aberrantly activated in most cancers of epithelial origin. The STAT3 signaling pathway plays a vital role in determining the outcome of the interaction between cancers and immune cells. It has the ability to induce a lot of genes that are critical for facilitating a tumor-promoting inflammatory microenvironment during tumor initiation and cancer progression [41]. Cigarette smoke extract may contribute to the activation of STAT3 phosphorylation and proinflammation cytokine secretion by inducing the negative regulation of CISH, the endogenous repressor of STAT3, by miR-944 [38].

#### 2.2.2. Oxidative Stress

Oxidative stress plays an important role in the pathogenesis of cigarette-smoke-induced COPD. Cigarette smoke exposure has been reported to increase the oxidative stress level of macrophages and promote the production of reactive oxygen species (ROS), which at higher concentrations is harmful to organisms, causing oxidative damage to most cellular components, including membrane lipids, enzymes, and DNA [42]. ROS and other oxidants can stimulate signal transduction pathways, such as nuclear factor erythroid-2-related factor-2 (Nrf2) and NF-κB signaling pathways, to alter cellular redox homeostasis. Cigarette smoke extract has been reported to mediate the expression of Nrf2 in human tracheal smooth muscle cells, and Nrf2 plays a protective role against oxidative stress through regulating antioxidative genes, such as NADPH quinone oxidoreductase 1 (NQO1) and heme oxygenase-1 (HO-1) [43].

#### 2.2.3. Cell Death

Cell death is the irreversible cessation of life phenomena and the end of life and is necessary to maintain tissue function and shape. Programmed cell death is required for the normal development and maintenance of tissue homeostasis and for eliminating damaged, infected, or superfluous cells in multicellular organisms [44]. Studies have shown that cigarette-smoke-induced damage is associated with multiple cell death mechanisms. Cigarette smoke extract can induce caspase-3-dependent apoptosis via endoplasmic reticulum stress and the intracellular Ca^2+^/p38/STAT1 pathway, leading to an increased risk of lung infection [45]. Necroptosis caused by cigarette smoke may contribute to the development of pulmonary emphysema via the p38 MAPK/RIPK3/MLKL pathway [46]. Pyroptosis, a type of cell death triggered by inflammasome activation, has a vital role in COPD progression. Cigarette smoke extract can induce pyroptosis through the ROS/NLRP3/caspase-1 pathway [47]. Exposure to cigarette smoke can induce labile iron accumulation and enhanced lipid peroxidation with concomitant nonapoptotic cell death, also known as ferroptosis, which is negatively regulated by GPx4 activity [48]. There are also reports that cigarette smoke exposure can induce cellular autophagy, and excessive autophagy can degrade cytoplasmic components, leading to autophagic cell death [49,50]. Exposure to cigarette smoke can activate the FOXO3-mediated autophagy pathway, resulting in increased expression of autophagy-related proteins (Beclin1, ATG5, ATG12, ATG16, and LC3B) [49].

#### 2.2.4. Epithelial–Mesenchymal Transition

Epithelial–mesenchymal transition (EMT) is a process that allows an epithelial cell to acquire a mesenchymal phenotype through multiple biochemical changes and is associated with an invasive or metastatic phenotype of cancer. It has been reported that cigarette smoke exposure could promote EMT in lung cancer cells through nAChR-dependent or TGF-β-dependent pathways [51,52]. In addition, cigarette smoke exposure can activate the WNT/β-catenin pathway to regulate the EMT programming in the airway epithelium in COPD [53].

#### 2.2.5. Genomic Instability

Cigarette smoke has been reported to increase the mutational burden and cancer risk, mainly due to the mix replication of DNA damage caused by cigarette carcinogens [54]. The maintenance of genomic stability has an important role in all organisms’ viability. Cigarette smoke can induce DNA damage response and genomic instability by promoting substantial inflammatory cell infiltration mediated by IL-7 in human bronchial epithelial cells [55]. In addition, noncoding RNAs are critical for maintaining genomic stability and directly regulate cellular processes involved in DNA damage response by changing their targeting genes’ expression [56]. It has been reported that lncRNA LCPAT1 is upregulated in DNA damage caused by cigarette smoke exposure by binding to RCC2 and increasing the stability and expression of RCC2 [57].

The above toxicity mechanisms are closely related to cigarette-induced COPD and lung cancer diseases. Cigarette smoke contains numerous chemical compounds, many of which can directly contribute to activating toxicity mechanisms. The main toxicants of cigarette smoke, such as aldehydes, N-nitrosamines, VOCs, PAHs, transition metals, and solid particulate matter, are associated with toxicity damage, including inflammation, oxidative stress, and DNA damage [17]. These toxicants are mainly produced by the incomplete combustion of tobacco. To reduce the harm of cigarettes, e-cigarettes that avoid tobacco burning and reduce the toxicity of smoking came into being.

## 3. Effects of E-Cigarettes on the Respiratory System

### 3.1. E-Cigarettes and Respiratory Diseases

Since the development of COPD and lung cancer is a long-term process, there is no report that e-cigarettes are directly associated with lung diseases, but studies have shown that acute e-cigarette exposure may cause various respiratory symptoms [58,59]. Current research studies suggest that the relationship between e-cigarettes and COPD is controversial. A 5-year prospective cohort survey of 10,294 subjects was conducted to determine whether e-cigarette use was associated with longitudinal changes in COPD progression. It was found that e-cigarette use among current conventional cigarette smokers with or at risk for COPD was associated with worse lung-related health outcomes [60]. In addition, exposure to nicotine-containing e-cigarette promotes cytokine expression, airway hyper-reactivity, and lung tissue destruction, all of which are associated with the development of COPD [61,62]. However, another study found that mice chronically exposed to e-cigarette smoke for 8 months did not develop a COPD phenotype [63].

Both active and passive smoking lead to an increased incidence of asthma. In addition, e-cigarette use is associated with an increased odd of self-reported asthma. People with asthma are currently more likely to use much higher amounts of smoke and vape products than those without asthma [64,65]. Based on investigation on the duration of immediate respiratory effects of e-cigarette smoking in mild asthmatics compared with healthy smokers, e-cigarette use had direct mechanical and inflammatory respiratory effects in healthy smokers and mild asthma smokers, but these changes were more prominent in the individuals with mild asthma [66]. However, there are also reports that, compared with conventional cigarette use, e-cigarette use can reverse harm from tobacco smoking in asthma patients and improves asthma conditions, and these beneficial effects may persist in the long term [67].

Although e-cigarettes may have certain harms, they appear safer than conventional cigarettes. Therefore, e-cigarettes may help reduce the risk of conventional cigarette-related diseases.

### 3.2. E-Cigarette-Related Toxicity Mechanisms

Similar to conventional cigarette, e-cigarette-related toxicity mechanisms are mainly related to inflammation response, oxidative stress, and DNA damage, and some new mechanisms have also received attention. Studies evaluate the toxicity mechanisms of e-cigarettes from multiple perspectives, such as in vivo and in vitro exposure levels, exposure duration, and exposure components. Here, we offer a detailed summary of the toxicity mechanisms of e-cigarettes (Table 2).

#### 3.2.1. Inflammation Response

E-cigarettes can induce inflammation response. According to reports, at the in vitro level, e-cigarette smoke acute exposure could promote the release of proinflammatory mediators (IL-6, IL-8, CXCL1, and G-CSF) to a lower degree than conventional cigarette smoke in primary normal human bronchial epithelial (NHBE) cells and BEAS-2B cells [68,69]. Similarly, acute exposure of human airway epithelial (H292) cells and human fetal lung fibroblasts (HFL1) in an air–liquid interface to e-cigarette aerosols resulted in increased secretion of IL-6 and IL-8, followed by morphological changes [70]. Scott et al. found that exposure of human alveolar macrophages from nonsmokers to e-cigarette aerosol condensate for 24 h revealed increased cytotoxicity and increased production of ROS, inflammatory cytokines, and chemokines, triggering an inflammatory state in alveolar macrophages within the lung [71]. Moreover, exposure to the Virginia tobacco- and menthol-flavored e-cigarette aerosols stimulated comparable inflammatory cytokine (IL-6 and IL-8) release to conventional cigarette smoke on bronchial epithelial cells (BECs) from patients with COPD [72]. In addition, at the in vivo level, exposure to e-cigarette aerosols can trigger inflammatory responses and adversely affect respiratory system mechanics in mice models [73]. Compared with other components (PG/VG or nicotine), tobacco-flavored, nicotine-containing e-cigarette aerosols had more significant promoting effects on levels of proinflammatory cytokines (TNF-α, IL-1β, IL-6) in mouse lung homogenates after both 3 days and 4 weeks of exposure [74]. Chronic inhalation of e-cigarette aerosols also increased the levels of circulating inflammatory cytokines after both 3 and 6 months of exposure in C57BL/6 and CD-1 mice [75]. E-cigarettes, their components, and exposure duration can all influence inflammatory responses.

#### 3.2.2. Oxidative Stress

Oxidative stress arises as a result of unbalanced levels of oxidants and antioxidants, with endogenous antioxidant defenses being impaired or overwhelmed by the presence of ROS [42]. E-cigarettes have been reported to not only promote inflammation response but also induce oxidative stress. E-cigarette aerosols can modulate oxidative stress markers in both lung cells and mouse lungs and activate the level of glutathione redox in vivo in the lung to induce oxidative stress [70].

Moreover, Lerner et al. found that acute e-cigarette aerosol exposure increased mitochondrial sensitivity in HFL-1 cells caused by elevated levels of mitochondrial ROS and reduced stability of an electron transport chain (ETC) complex IV subunit, thus inducing mitochondrial stress [76]. E-cigarette aerosols showed relatively low levels of oxidative stress compared with cigarette smoke, but increasing e-cigarette power and exposure times could stimulate elevated levels of related oxidative stress [10]. In addition, PG and VG, two common e-cigarette solvent carriers, can induce oxidative stress at the in vivo level by significantly increasing the levels of 8-hydroxy-2′-deoxyguanosine (8-OHdG), a biomarker of DNA oxidative damage, in the lung and plasma of B6C3F1 mice after 8 weeks of exposure. In contrast, exposure to PG/VG-containing nicotine declined the levels of 8-OHdG [77]. It has been reported that nicotine has free radical scavenging properties and might have neuroprotective effects [92]. The effect of flavors on e-cigarette-induced oxidative stress in pulmonary cells has also been reported. Comparing six popular flavors of e-cigarettes ((tobacco, fruity, minty fruit, minty/cool (iced), drinks/beverages, and desserts), ROS generated from e-cigarette bars varies significantly among different flavors and flavors of varying nicotine contents [78]. Another study found that menthol flavor e-cigarette exposure to BEAS-2B cells caused mitochondrial dysfunction, reducing basal and maximal respiration, consequently affecting mitochondrial oxidative stress [79]. Moreover, cinnamon-flavored e-cigarette aerosols could induce ROS production and greater cytotoxicity than flavorless aerosol [80]. Cinnamaldehyde has been identified as the most potent chemical in cinnamon-flavored e-liquids, which can cause oxidative stress in lung cells [81]. The above reports confirm that both e-cigarettes and their main ingredients affect oxidative stress.

#### 3.2.3. DNA Damage

DNA is only sufficiently stable to serve as the carrier of genetic information and needs to repair damage accurately to maintain its integrity. DNA damage originates from two sources, endogenous sources, such as hydrolysis and oxidative damage, and environmental sources, such as external physical radiation and tobacco smoke [93]. E-cigarettes can also cause DNA damage. It has been reported that e-cigarette aerosol exposure-induced mitochondrial ROS production could cause nuclear DNA fragmentation in HFL-1 cells [76]. E-cigarette aerosol extract also induced DNA damage in a dose-dependent manner without dependence on nicotine concentration and decreased the levels of proteins essential for the removal of DNA damage, such as OGG1 and ERCC1 [77,82]. Similarly, it could induce ROS, cause DNA damage and apoptosis, and significantly reduce cell viability in a dose-dependent manner [83]. In addition, at the in vivo level, e-cigarette aerosols not only induced mutagenic γ-OH-PdG and O6-MedG adducts in FVBN mouse lung, bladder, and heart, but also inhibited DNA repair and reduced the repair proteins XPC and OGG1/2 in lung tissue [84]. E-cigarette aerosols could damage DNA by increasing oxygen-free radical production and DNA oxidation and consequently cause comutagenic and cancer-initiating effects in SD rat lung models [85]. Hence, e-cigarette aerosols have the potential to enhance mutation susceptibility and induce neoplastic transformation.

#### 3.2.4. Other Mechanisms

In addition to the above three mechanisms, some studies have also found that new toxicity mechanisms of e-cigarettes involve cell apoptosis, EMT, and transcriptomic changes. E-cigarette aerosol condensate can induce cell apoptosis [71,83]. It has been reported that e-cigarette aerosol exposure could induce cell apoptosis, thereby reducing the rate of human gingival fibroblast proliferation, with or without nicotine [86]. Furthermore, e-cigarette aerosols increased the percentages of apoptotic/necrotic human gingival epithelial cells with an increasing number of exposures and were associated with the caspase-3 pathway [87]. EMT is the initiating pathway of cancer cell metastasis. It has been found that menthol or tobacco-flavored e-cigarette liquids or aerosols induced EMT of A549 cells after 3–8 days of long-term exposure and caused cells to acquire fibroblast-like morphology, loss of intercellular junctions, internalization of E-cadherin, increased motility, and upregulation of EMT markers [88]. In addition, exposure to e-cigarette aerosols caused significant transcriptomic changes related to ribosomal protein synthesis in primary human airway epithelial cells. Ribosome biogenesis and protein translation are essential for cell growth, proliferation, differentiation, and development [89]. Studies have also shown that e-cigarette aerosol exposure had a profound effect on gene expression, with differential expression of miRNAs, lncRNAs, and mRNAs after e-cigarette treatment [90,91].

In conclusion, current studies have shown that the toxicity mechanisms of e-cigarettes involve multiple aspects, including inflammatory responses, oxidative stress, DNA damage, apoptosis, EMT, and transcriptomic changes. Although e-cigarettes do not contain tobacco and produce significantly fewer toxic substances than conventional cigarettes, e-cigarette liquid is a mixed liquid, and the component after atomization can affect the toxicity mechanism of e-cigarettes. Extensive research studies are still needed to explore the potential and detailed toxicity mechanisms of e-cigarettes.

### 3.3. E-Cigarette-Related Signal Pathways

#### 3.3.1. MAPK Signal Pathway

Recently, the MAPK signal pathway has been proposed to play a key role in the inflammatory response induced by e-cigarettes. Exposure to e-cigarette aerosol extract caused proinflammatory responses in human neutrophils by increasing matrix metalloproteinase-9 (MMP-9) and chemokine C-X-C motif ligand 8 (CXCL8) secretion and upregulating MMP-9 and neutrophil elastase (NE) activity. MMP-9 and NE activity is positively correlated with COPD severity. These proinflammatory changes were accompanied by p38 MAPK activation with the phosphorylation of p38, which may cause pulmonary inflammation [94]. Similarly, exposure to e-cigarette aerosol extract also induced IL-6 production on human dendritic cells by activating the MAPK pathway with the increase in the phosphorylation level of p38 and ERK1/2 [95].

#### 3.3.2. NK-κB Signal Pathway

The expression of proinflammatory mediators is also regulated by the NF-κB signal pathway. E-cigarette aerosol condensate mediated inflammation in A549 cells with significant upregulation of cytokines and chemokines (IL-6, IL-8, and MCP-1). These inflammatory effects were mediated by molecular interactions between membrane-bound toll-like receptor 4 (TLR-4) and cytosolic receptor nucleotide-binding oligomerization domain-containing protein-1 (NOD-1) with the lipid raft-associated protein caveolin-1. This complex resulted in the degradation of I_K_Bα and the activation of transcription factor NF-κB. Activated NF-κB translocated to the nucleus for binding to the consensus sequences of target genes and consequently induced inflammatory response [96].

#### 3.3.3. Nrf2 Signal Pathway

It has been found that E-cigarette aerosol exposure induced an increase in mitochondrial ROS and increased the expression of NQO1. The upregulation of the antioxidant response element protein NQO1 suggested activating the Nrf2 pathway [76]. Similarly, exposure of primary human bronchial epithelial (NHBE) cells to e-cigarette liquid resulted in the induction of oxidative stress-response genes, including GCLM, GCLC, GPX2, NQO1, and HO-1, which suggested that e-cigarettes can activate the Nrf2 signaling pathway through oxidative stress [90]. Moreover, the activation of the Nrf2 signal pathway, through its action as a transcription factor, upregulates the superoxide dismutases SOD2 or SOD1, which could reduce lung damage caused by smoking [97]. Both conventional cigarette and e-cigarette exposure elevated the expression of Nrf2 and SOD1 proteins in mice models, suggesting that e-cigarette exposure did cause oxidative stress by activating the Nrf2 pathway to reduce damage [98]. In addition, e-cigarette aerosol exposure promoted oxidative stress (ROS, HO-1, 3-nitrotyrosine (3-NT), and 4-hydroxynonenal (4-HNE)), inflammation (IL-6 and CD68), and endothelial dysfunction by activating phagocytic NADPH oxidase (NOX-2). Toxic aldehydes, such as acrolein, were generated during e-cigarette vaporization as key mediators of these adverse consequences [99].

#### 3.3.4. PKCα/ERK Signal Pathway

Protein kinase C-α (PKC-α) has been involved in a broad range of cellular functions. The ubiquitous expression and activation by various stimuli implicate it in many cellular functions, including differentiation, proliferation, apoptosis, cellular transformation, motility, adhesion, and so on [100]. It has been found that inhalation of nicotine in e-cigarette aerosols activated the PKCα/ERK signal pathway by increasing the phosphorylation of PKCα and ERK through the nicotinic receptors α7nAchRs. The activated PKCα/ERK signal pathway then induced lung cytokine (IL-1β, Il-6, MCP-1, and CXCL2) and protease (MMP-9, MMP-12) expression and consequently regulated lung inflammation in the mouse lung [61].

#### 3.3.5. Ca^2+^ Signal Pathway

Cytoplasmic Ca^2+^ is a key second messenger that can control many cellular functions. Dysregulation of cell Ca^2+^ homeostasis is linked to several disease processes, including autoimmune disease, cancer, inflammation, and neurodegeneration [101]. Ghosh et al. exposed HEK293T cells and THP-1 macrophage-like cells to different JUUL e-liquids and found that e-liquids caused significant cytotoxic effects and a significant elevation in cytoplasmic Ca^2+^. Among the several flavors, mint e-liquid caused the most rapid increase in cytoplasmic Ca^2+^ in both cell types, with the underlying mechanism that mint flavor inhibited endoplasmic reticulum (ER)-mediated Ca^2+^ transport and caused ER Ca^2+^ depletion [102]. Moreover, another study found that chronic nicotine-containing e-cigarette vapor exposure can increase cytoplasmic Ca^2+^ via the nicotinic acetylcholine receptors nAChRs to induce MMP protease release from bronchoalveolar lavage macrophages and peripheral blood neutrophils and further disrupt the protease–antiprotease balance and increase risk of lung damage [103].

#### 3.3.6. AhR Signal Pathway

Persistent activation of the aryl hydrocarbon receptor (AhR) is thought to be a risk factor for carcinogenesis. It has been reported that AhR played a key role in the nicotine-induced cytochrome P-450 1A1 (CYP1A1) expression [104]. CYP1A1 was an important enzyme activated by numerous xenobiotics, such as polycyclic aromatic hydrocarbon carcinogens and nicotine. The study indicated that nicotine significantly stimulated the expression of CYP1A1 mRNA dependent on the AhR signal way, which triggered cancer cell proliferation. Similarly, e-cigarette aerosol condensate promoted the metabolism of the tobacco carcinogen benzo(a)pyrene (BaP) to genotoxic products in human oral keratinocyte cell. Conversion of BaP to its genotoxin required the induction of CYP1A1 and CYP1B1, likely via the activation of the AhR [105].

#### 3.3.7. EGFR Signal Pathway

It has been reported that e-liquid exposure can promote cell proliferation and malignancy of brain tumors by activating the epidermal growth factor receptor (EGFR) associated with cell growth. E-liquid exposure induced an increased level of EGFR phosphorylation in a dose-dependent manner in brain cancer cells and further induced the increased expression of ERK phosphorylation, a downstream effector of the EGFR signaling pathway [106]. Thus, e-liquid activated EGFR signaling and affected cell growth via pERK activation.

**Figure 3:**
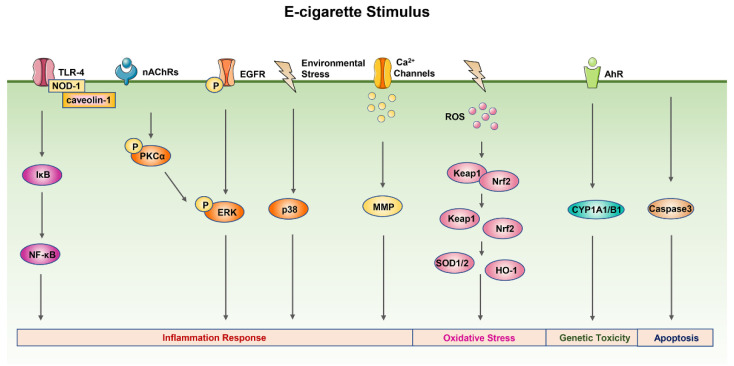
The signal pathways of e-cigarette toxicity mechanisms. E-cigarette smoke exposure can also activate the p38 MAPK, TLR-4/NF-κB, and EGFR/ERK signal pathways to promote the release of inflammatory mediators. The PKCα/ERK pathway is stimulated by e-cigarette smoke through the nicotinic receptors α7nAchR to cause inflammation response. E-cigarette smoke exposure can increase cytoplasmic Ca^2+^ to induce MMP protease release and increase the risk of lung damage. In addition, the activation of the Nrf2 signal pathway caused by e-cigarette smoke exposure following the upregulation of SOD and HO-1 could reduce lung damage. E-cigarette smoke exposure can also cause genetic toxicity via the AhR signal pathway’s activation and cell apoptosis via the caspase-3 signal pathway. Abbreviation: AhR, aryl hydrocarbon receptor; CYP1A1/B1; cytochrome P-450 1A1/B1; EGFR, epidermal growth factor receptor; ERK, extracellular regulated protein kinases; MMP, matrix metalloproteinase; NOD-1, nucleotide-binding oligomerization domain-containing protein-1; PKCα, protein kinase C-α; SOD, superoxide dismutase; TLR-4, toll-like receptor 4.

Here, we offer a detailed schematic of the signal pathways of e-cigarette toxicity mechanisms (Figure 3).

## 4. Conclusions and Perspectives

E-cigarettes do not contain tobacco, and the ingredients are far fewer than those of conventional cigarettes. The harmful substances produced by heating atomization are also fewer than those of tobacco burning. Although the harm of e-cigarettes is much lower than that of conventional cigarettes, the health risks posed by e-cigarettes cannot be ignored. This article summarizes the existing literature reports on the toxicity mechanism of e-cigarettes and briefly compares the differences in the toxicity mechanisms of e-cigarettes and conventional cigarettes. It was revealed that the toxicity mechanism and related signal pathways of e-cigarettes were similar to those of cigarettes, such as inflammation response, oxidative stress, and DNA damage. Additionally, since e-cigarettes have been on the market for less than 20 years, most studies have assessed the short-term effects of e-cigarettes based on acute exposure. Little is known about chronic effects, especially in humans.

With the widespread use and the popularity of e-cigarettes among adults, the Food and Drug Administration (FDA), on 6 February 2020, implemented a policy for unauthorized flavored prefilled pod or cartridge-based e-cigarettes, which applies to all flavors with nicotine, excluding menthol and tobacco. However, all flavored products without nicotine are still available in the market [107]. Although not all e-cigarettes contain flavors, there are few studies on flavor-related mechanisms. Therefore, it is necessary to explore the safety and toxicity mechanisms of flavoring agents in e-cigarettes. The potential health risks brought by different flavors of e-cigarettes cannot be ignored. Moreover, the presence of different e-liquid constituents can induce potential toxic effects on pulmonary health [108]. In addition to considering the toxicity of e-cigarette liquids or aerosols alone, a comparative analysis of the single components of e-cigarettes to comprehensively evaluate the overall safety of e-cigarettes can also be an aspect of future study.

At present, many studies on the toxicity mechanism of e-cigarettes are carried out on normal cells or tool cells. However, patients with lung diseases often smoke, and e-cigarettes have also been chosen as a substitute for conventional cigarettes. The potential impact of e-cigarette use on the safety of patients with lung disease needs to be considered. Therefore, it is still necessary to consider the effects of e-cigarettes on the mechanism of lung-disease-related cells, such as lung cancer cells or patient-derived cells. Further comparison of the differences in the mechanisms of toxicity between e-cigarettes and cigarettes is required.

The current research mainly focuses on the lung effects of e-cigarettes in in vitro and in vivo models and the exploration of the mechanisms of inflammatory response and oxidative stress. Although it is very informative, after lung inhalation of e-cigarettes into the systemic blood circulation, it can still cause potential damage to other organ systems, such as the cardiovascular system and immune system, and induce different physiological mechanisms. Thus, more scientific data are needed to provide evidence-based information about the toxicology mechanisms of e-cigarettes to help people view e-cigarettes’ use objectively and rationally.

At the same time, there are differences in the regulation of e-cigarettes between countries. The United States government banned the sale of flavored e-cigarettes in 2020. On the other hand, the United Kingdom supports e-cigarettes to quit smoking. As e-cigarettes are very popular among young people, the government should strengthen strict supervision of e-cigarettes, limit their availability, and prevent young people and nonsmokers from abusing e-cigarettes. Moreover, e-cigarette quality standards and e-cigarette toxicity evaluation systems should be established to standardize various components in e-cigarettes, not only to prevent the harm of counterfeit e-cigarettes to people, but also to standardize and effectively evaluate the toxicity of e-cigarette products. However, what is more important is to raise people’s awareness of the correct use of e-cigarette products and not ignore the potential health risks of e-cigarettes.

## Figures and Tables

**Table 1:** Differences in toxic compounds in cigarette and e-cigarette smoke [17,18,19].

Toxic Compound Type	Toxic Compound	Concentration Range Cigarette (/Puff)	Concentration Range E-Cigarette (/Puff)
Carbonyls	Formaldehyde	<10 µg	<82 µg
	Acetaldehyde	<140 µg	<53 µg
	Acrolein	<14 µg	<3.3 µg
	Propionaldehyde	<5.9 µg	<1.79 µg
	Crotonaldehyde	<2 µg	<0.04 µg
N-nitrosamines	N’-nitrosonornicotine (NNN)	<370 ng	<0.029 ng
	N’-nitrosoanabasine (NAB)	<15 ng	<0.01 ng
	4-(methylnitrosamino)-1-(3-pyridyl)-1-butanone (NNK)	<77 ng	<0.019 ng
	N’-nitrosoanatabine (NAT)	<16 ng	<0.085 ng
Volatile organic compounds (VOCs)	Toluene	<6.9 µg	<1.53 µg
	Benzene	<4.5 µg	<0.41 µg
Inorganic compounds	Nickel	<60 ng	<6.4 ng
	Cobalt	<0.02 ng	<0.58 ng
	Chromium	<7 ng	<9 ng
	Lead	<8.5 ng	<3.8 ng
	Cadmium	<35 ng	-
	Zinc	<1370 ng	<458 ng
	Cuprum	<130 ng	<20.9 ng
	Carbon monoxide (CO)	<2.3 mg	-
Polycyclic aromatic hydrocarbons and heterocyclic aromatic hydrocarbons (PAHs)	Benz[a]anthracene	<7 ng	-
	Benzo[b + k]fluoranthene	<3.4 ng	-
	Benzo[a]pyrene	<4 ng	-
	Dibenzo[a, h]anthracene	<0.4 ng	-
Nicotine		<0.3 mg	<0.142 mg
Particulate matter	Total particulate matter (TPM)	<1.7 mg	<5.8 mg

**Table 2:** Studies of the toxicity mechanisms of e-cigarette.

Toxicity Mechanism	Cells/Animals	E-Cigarette Model	Exposure Method	Toxicity Findings	Reference
Inflammation response Oxidative stress	NHBE cells Human 3D bronchial epithelial tissue	PG/VG: 70:30 Nicotine: 2% Flavors: flavorless	Incubation with media containing e-liquids for 24 h	Decreased cell viability, increase in G-CSF, CXCL1, and IL-8 and levels of GSH and ROS	[68]
Inflammation response	BEAS-2B cells	Lounge model designed with 2.8 Ω coil and 3.6 V power supply PG/VG: 65:35 Nicotine: 0 and 16 mg/mL Flavors: blond tobacco, chlorophyll mint, and unflavored	Air–liquid interface for 8 or 48 min (35 mL puff volume, 2 s draw, 60 s puff interval)	Low increase in IL-6	[69]
Inflammation response Oxidative stress	H292 cells HFL1 cells C57BL/6J mice	Refillable ENDS with 2.2 Ω Nicotine: 0 and 16 mg/mL Flavors: classic tobacco, cinnamon roll, grape vape, American tobacco, etc.	Air–liquid interface for 5, 10, and 15 min (a puff of 3–4 s, 30 s puff interval)	Decreased cell viability, increase in IL-6 and IL-8, promotion of OX/ROS generation, and lung inflammation in mice	[70]
Inflammation response Oxidative stress Apoptosis	Human alveolar macrophages	Second-generation END with 650 mAh battery and 1.8 Ω coil PG/VG: 50:50 Nicotine: 0 and 36 mg/mL Flavors: flavorless	Incubation with media containing e-cigarette vapor condensate for 24 h	Decreased cell viability, increased apoptosis, increased ROS production and levels of IL-6, TNF-α, CXCL8, MCP-1, and MMP-9	[71]
Inflammation response DNA damage	Bronchial epithelial cells 16HBE cells	JUUL^®^ e-cigarette Nicotine: 5% Flavors: Virginia tobacco and menthol	Air–liquid interface for 30 min (55 mL puff volume, 4 s draw, 30 s puff interval)	Decreased cell viability, increase in IL-6, IL-8, and 8-OHdG	[72]
Inflammation response	BALB/c mice	Four different varieties of e-cigarette (Mt. Baker Vapor, Lynden, WA, USA) Nicotine: 0 and 12 mg/mL Flavors: American Tobacco	Whole-body exposure for 8 weeks	Increase in pulmonary inflammation and responsiveness to methacholine	[73]
Inflammation response	C57BL/6J mice	PG/VG: 1:1 Nicotine: 0 and 18 mg/mL Flavors: tobacco blend	Whole-body exposure for 3 days or 4 weeks	Increase in BALF cellularity, levels of IL-1β, IL-6, pulmonary inflammation, and responsiveness to methacholine	[74]
Inflammation response	C57BL/6 mice CD-1 mice	E-liquid was placed in a standard tank (1.8 Ω) with a rechargeable battery (3.4 V) PG/VG: 50:50 Nicotine: 24 mg/mL Flavors: flavorless	Nose-only inExpose system exposure for 3–6 months	Increase in circulating inflammatory cytokines	[75]
Oxidative stress DNA damage	HFL-1 cells	Lorillard Blu Classic Tobacco E-cigarette Nicotine: 16 mg/mL Flavors: classic tobacco	Air–liquid interface for 5, 10, 15, or 20 min (a puff of 3–4 s, 30 s puff interval)	Increase in mtROS, nuclear DNA fragmentation, and decrease in stability of an electron transport chain (ETC) complex IV subunit	[76]
Oxidative stress	BEAS-2B cells	Second-generation “Lounge” model with a 2.8 Ω nichrome coil and 4.6 W power supply and third-generation “ModBox” model with a 0.5 Ω Kanthal coil PG/VG: 65:35 Nicotine: 16 mg/mL Flavors: blond tobacco	Air–liquid interface for 40, 80, and 120 puffs (55 mL puff volume, 2 s draw, 30 s puff interval)	Increase of GSSG/GSH ratio at higher power settings	[10]
Oxidative stress DNA damage	B6C3F1 mice	PG/VG: 1:1 Nicotine: 0, 12, and 24 mg/mL Flavors: flavorless	Whole-body exposure for 8 weeks	Increase in 8-OHdG	[77]
Oxidative stress	Acellular ROS assay	PG/VG: 1:1 Nicotine: 0%–6.8% Flavors: tobacco, minty fruit, fruity, minty/cool (iced), desserts, and drinks/beverages	Incubation with media containing of e-cigarette vapor condensate for 15 min	Increase in ROS	[78]
Oxidative stress	BEAS-2B cells	JUUL^®^ pod Nicotine: 5% Flavors: menthol and Virginia tobacco	Air–liquid interface for 30 min (55 mL puff volume, 3 s draw, 30 s puff interval)	Change of mitochondrial bioenergetics and decrease in mitochondrial respiration	[79]
Oxidative stress	MG-63 cells	Mister-E-Liquid and Vape Dudes PG/VG: 1:1 Nicotine: 0 mg/mL Flavors: flavorless and cinnamon	Incubation with media containing e-cigarette vapor condensate for 24 or 48 h	Decreased cell viability, increase in ROS	[80]
Oxidative stress Inflammation response	U937 cells Mono Mac 6 cells	Nicotine: 0 mg/mL Flavors: strawberry zing, café latte, pineapple coconut, cinnamon roll, etc.	Incubation with media containing of e-cigarette vapor condensate for 24 h	Decreased cell viability, increase in ROS and IL-8	[81]
DNA damage	Human epithelial normal bronchial cells (Nuli1) Human premalignant dysplastic oral mucosal keratinocyte cells (POE9n)	Brands NJoy and eGo-T PG/VG: 50:50 Nicotine: 0, 12, and 18 mg/mL Flavors: traditional tobacco and desert sands	Incubation with media containing of e-cigarette vapor condensate for 2 weeks (1 h per day)	Increase in 8-oxo-dG and ROS, decrease in the expression of ERCC1 and OGG1	[82]
DNA damage Oxidative stress Apoptosis	HUVEC cells	Brands Blu, Vuse, Green Smoke, and NJoy Nicotine: 2.4%, 4.5%, and 4.8% Flavors: tobacco	Incubation with media containing e-cigarette vapor condensate for 24 or 72 h	Decreased cell viability, increase in DNA damage, apoptosis, and ROS	[83]
DNA damage	BEAS-2B cells UROtsa cells FVBN mice	Brand NJoy PG/VG: 50:50 Nicotine: 10 mg/mL	Whole-body exposure for 12 weeks and cell exposure for 1 h	Increase in γ-OH-PdG and O6-MedG, decrease in the expression of repair proteins XPC and OGG1/2	[84]
DNA damage	Sprague Dawley rats	Brand Essential cloud with a 2000 mAh battery and 2 Ω coil Nicotine: 18 mg/mL Flavors: red fruit	Whole-body exposure for 4 weeks	Increase in the free radical content, 8-OHdG, and DNA fragmentation	[85]
Apoptosis	Human primary gingival fibroblasts	Brand EMOW Nicotine: 12 mg/mL Flavors: smooth Canadian tobacco	Incubation with media containing e-cigarette vapor condensate for 24 h	Decrease in cell density and altered cell morphology, increase in cell apoptosis	[86]
Apoptosis	Human primary gingival epithelial cells	Brand EMOW Nicotine: 12 mg/mL Flavors: smooth Canadian tobacco	Air–liquid interface for 1, 2, or 3 days with 15 min per day (5 s draw, 30 s puff interval)	Increase in cell apoptosis and caspase-3 activity	[87]
Epithelial–mesenchymal transition	A549 cells	Nicotine: 48 mg/mL Flavors: menthol and tobacco	Incubation with media containing e-cigarette vapor condensate for 3–4 days	Acquisition of a fibroblast-like morphology, loss of cell-to-cell junctions, internalization of E-cadherin, increased motility, and upregulation of EMT markers	[88]
Transcriptomic changes	NHBE cells	No mention	Air–liquid interface for 6 or 24 h	Inducement of significant transcriptomic changes, increase in expression of ribosomal protein genes, change of ribosomal RNA transcription and protein synthesis	[89]
Transcriptomic changes	NHBE cells	E-cigarette liquid Nicotine: 0% or 2.4%	Incubation with media containing e-cigarette vapor condensate for 48 h	Change of microRNA expression profiling and increase in expression of multiple miRNAs	[90]
Transcriptomic changes	iPSC-EC cells	Vape Dudes E-cigarette PG/VG: 50:50 Nicotine: 24 mg/mL Flavors: menthol	Incubation with media containing e-cigarette vapor condensate for 24 h	Change of expression profiling of lncRNAs and mRNAs	[91]

## Data Availability

Not applicable.

## References

[1] Yamin C.K., Bitton A., Bates D.W. (2010). E-Cigarettes: A Rapidly Growing Internet Phenomenon. Ann. Intern. Med..

[2] Sapru S., Vardhan M., Li Q., Guo Y., Li X., Saxena D. (2020). E-cigarettes use in the United States: Reasons for use, perceptions, and effects on health. BMC Public Health.

[3] Protano C., Avino P., Manigrasso M., Vivaldi V., Perna F., Valeriani F., Vitali M. (2018). Environmental Electronic Vape Exposure from Four Different Generations of Electronic Cigarettes: Airborne Particulate Matter Levels. Int. J. Environ. Res. Public Health.

[4] Barrington-Trimis J.L., Leventhal A.M. (2018). Adolescents’ Use of “Pod Mod” E-Cigarettes—Urgent Concerns. N. Engl. J. Med..

[5] Gentzke A.S., Wang T.W., Jamal A., Park-Lee E., Ren C., Cullen K.A., Neff L. (2020). Tobacco Product Use among Middle and High School Students—United States, 2020. MMWR Morb. Mortal. Wkly. Rep..

[6] Bauld L., MacKintosh A.M., Eastwood B., Ford A., Moore G., Dockrell M., Arnott D., Cheeseman H., McNeill A. (2017). Young People’s Use of E-Cigarettes across the United Kingdom: Findings from Five Surveys 2015–2017. Int. J. Environ. Res. Public Health.

[7] Ahluwalia I.B., Smith T., Arrazola R.A., Palipudi K.M., Garcia D.Q.I., Prasad V.M., Commar A., Schotte K., Garwood P.D., Armour B.S. (2018). Current Tobacco Smoking, Quit Attempts, and Knowledge About Smoking Risks Among Persons Aged ≥15 Years—Global Adult Tobacco Survey, 28 Countries, 2008–2016. MMWR Morb. Mortal. Wkly. Rep..

[8] Zhao Z., Zhang M., Wu J., Xu X., Yin P., Huang Z., Zhang X., Zhou Y., Zhang X., Li C. (2020). E-cigarette use among adults in China: Findings from repeated cross-sectional surveys in 2015–2016 and 2018–2019. Lancet Public Health.

[9] Romijnders K., van Osch L., de Vries H., Talhout R. (2018). Perceptions and Reasons Regarding E-Cigarette Use among Users and Non-Users: A Narrative Literature Review. Int. J. Environ. Res. Public Health.

[10] Dusautoir R., Zarcone G., Verriele M., Garçon G., Fronval I., Beauval N., Allorge D., Riffault V., Locoge N., Lo-Guidice J. (2021). Comparison of the chemical composition of aerosols from heated tobacco products, electronic cigarettes and tobacco cigarettes and their toxic impacts on the human bronchial epithelial BEAS-2B cells. J. Hazard. Mater..

[11] Konstantinou E., Fotopoulou F., Drosos A., Dimakopoulou N., Zagoriti Z., Niarchos A., Makrynioti D., Kouretas D., Farsalinos K., Lagoumintzis G. (2018). Tobacco-specific nitrosamines: A literature review. Food Chem. Toxicol..

[12] Kosmider L., Kimber C.F., Kurek J., Corcoran O., Dawkins L.E. (2018). Compensatory Puffing with Lower Nicotine Concentration E-liquids Increases Carbonyl Exposure in E-cigarette Aerosols. Nicotine Tob. Res..

[13] Zhao D., Aravindakshan A., Hilpert M., Olmedo P., Rule A.M., Navas-Acien A., Aherrera A. (2020). Metal/Metalloid Levels in Electronic Cigarette Liquids, Aerosols, and Human Biosamples: A Systematic Review. Environ. Health Perspect..

[14] Helen G.S., Liakoni E., Nardone N., Addo N., Jacob P.R., Benowitz N.L. (2020). Comparison of Systemic Exposure to Toxic and/or Carcinogenic Volatile Organic Compounds (VOC) during Vaping, Smoking, and Abstention. Cancer Prev. Res..

[15] Flora J.W., Meruva N., Huang C.B., Wilkinson C.T., Ballentine R., Smith D.C., Werley M.S., McKinney W.J. (2016). Characterization of potential impurities and degradation products in electronic cigarette formulations and aerosols. Regul. Toxicol. Pharmacol..

[16] Olmedo P., Goessler W., Tanda S., Grau-Perez M., Jarmul S., Aherrera A., Chen R., Hilpert M., Cohen J.E., Navas-Acien A. (2018). Metal Concentrations in e-Cigarette Liquid and Aerosol Samples: The Contribution of Metallic Coils. Environ. Health Persp..

[17] Münzel T., Hahad O., Kuntic M., Keaney J.F., Deanfield J.E., Daiber A. (2020). Effects of tobacco cigarettes, e-cigarettes, and waterpipe smoking on endothelial function and clinical outcomes. Eur. Heart J..

[18] Gray N., Halstead M., Valentin-Blasini L., Watson C., Pappas R.S. (2022). Toxic Metals in Liquid and Aerosol from Pod-Type Electronic Cigarettes. J. Anal. Toxicol..

[19] Soleimani F., Dobaradaran S., De-la-Torre G.E., Schmidt T.C., Saeedi R. (2022). Content of toxic components of cigarette, cigarette smoke vs. cigarette butts: A comprehensive systematic review. Sci. Total Environ..

[20] Wang G., Liu W., Song W. (2019). Toxicity assessment of electronic cigarettes. Inhal. Toxicol..

[21] Merecz-Sadowska A., Sitarek P., Zielinska-Blizniewska H., Malinowska K., Zajdel K., Zakonnik L., Zajdel R. (2020). A Summary of In Vitro and In Vivo Studies Evaluating the Impact of E-Cigarette Exposure on Living Organisms and the Environment. Int. J. Mol. Sci..

[22] Wang L., Wang Y., Chen J., Yang X., Jiang X., Liu P., Li M. (2021). Comparison of biological and transcriptomic effects of conventional cigarette and electronic cigarette smoke exposure at toxicological dose in BEAS-2B cells. Ecotoxicol. Environ. Safe..

[23] US Centers for Disease and Control Prevention, US National Center for Chronic Disease Prevention and Health Promotion, US Office on Smoking and Health (2010). How Tobacco Smoke Causes Disease: The Biology and Behavioral Basis for Smoking-Attributable Disease: A Report of the Surgeon General.

[24] Tsai M., Byun M.K., Shin J., Crotty A.L. (2020). Effects of e-cigarettes and vaping devices on cardiac and pulmonary physiology. J. Physiol..

[25] Hou W., Hu S., Li C., Ma H., Wang Q., Meng G., Guo T., Zhang J. (2019). Cigarette Smoke Induced Lung Barrier Dysfunction, EMT, and Tissue Remodeling: A Possible Link between COPD and Lung Cancer. Biomed Res. Int..

[26] GBD 2015 Mortality and Causes of Death Collaborators (2016). Global, regional, and national life expectancy, all-cause mortality, and cause-specific mortality for 249 causes of death, 1980-2015: A systematic analysis for the Global Burden of Disease Study 2015. Lancet.

[27] Rabe K.F., Watz H. (2017). Chronic obstructive pulmonary disease. Lancet.

[28] Dransfield M.T., Kunisaki K.M., Strand M.J., Anzueto A., Bhatt S.P., Bowler R.P., Criner G.J., Curtis J.L., Hanania N.A., Nath H. (2017). Acute Exacerbations and Lung Function Loss in Smokers with and without Chronic Obstructive Pulmonary Disease. Am. J. Respir. Crit. Care Med..

[29] Gibbs K., Collaco J.M., McGrath-Morrow S.A. (2016). Impact of Tobacco Smoke and Nicotine Exposure on Lung Development. Chest.

[30] Alavinezhad A., Boskabady M.H. (2018). The prevalence of asthma and related symptoms in Middle East countries. Clin. Respir. J..

[31] Santos V., Moreira M., Rosa A., Sobragi S.M., Silva C., Dalcin P. (2022). Association of quality of life and disease control with cigarette smoking in patients with severe asthma. Braz. J. Med. Biol. Res..

[32] Ross K.R., Gupta R., DeBoer M.D., Zein J., Phillips B.R., Mauger D.T., Li C., Myers R.E., Phipatanakul W., Fitzpatrick A.M. (2020). Severe asthma during childhood and adolescence: A longitudinal study. J. Allergy Clin. Immunol..

[33] Grabenhenrich L.B., Gough H., Reich A., Eckers N., Zepp F., Nitsche O., Forster J., Schuster A., Schramm D., Bauer C.P. (2014). Early-life determinants of asthma from birth to age 20 years: A German birth cohort study. J. Allergy Clin. Immunol..

[34] Wang B., Chen H., Chan Y.L., Wang G., Oliver B.G. (2020). Why Do Intrauterine Exposure to Air Pollution and Cigarette Smoke Increase the Risk of Asthma?. Front. Cell Dev. Biol..

[35] Mortaz E., Adcock I.M., Ito K., Kraneveld A.D., Nijkamp F.P., Folkerts G. (2010). Cigarette smoke induces CXCL8 production by human neutrophils via activation of TLR9 receptor. Eur. Respir. J..

[36] Tao Y., Sun Y., Wu B., Xu D., Yang J., Gu L., Du C. (2021). Overexpression of FOXA2 attenuates cigarette smoke-induced cellular senescence and lung inflammation through inhibition of the p38 and Erk1/2 MAPK pathways. Int. Immunopharmacol..

[37] Qiu J.F., Ma N., He Z.Y., Zhong X.N., Zhang J.Q., Bai J., Deng J.M., Tang X.J., Luo Z.L., Huang M. (2021). Erythromycin inhibits cigarette smoke-induced inflammation through regulating the PPARγ/NF-κB signaling pathway in macrophages. Int. Immunopharmacol..

[38] Peng H.Y., Hsiao J.R., Chou S.T., Hsu Y.M., Wu G.H., Shieh Y.S., Shiah S.G. (2020). MiR-944/CISH mediated inflammation via STAT3 is involved in oral cancer malignance by cigarette smoking. Neoplasia.

[39] Xu L., Li X., Wang H., Xie F., Liu H., Xie J. (2019). Cigarette smoke triggers inflammation mediated by autophagy in BEAS-2B cells. Ecotoxicol. Environ. Saf..

[40] Schuliga M. (2015). NF-kappaB Signaling in Chronic Inflammatory Airway Disease. Biomolecules.

[41] Jarnicki A., Putoczki T., Ernst M. (2010). Stat3: Linking inflammation to epithelial cancer—More than a “gut” feeling?. Cell Div..

[42] Barnes P.J. (2020). Oxidative stress-based therapeutics in COPD. Redox Biol..

[43] Dang X., He B., Ning Q., Liu Y., Guo J., Niu G., Chen M. (2020). Alantolactone suppresses inflammation, apoptosis and oxidative stress in cigarette smoke-induced human bronchial epithelial cells through activation of Nrf2/HO-1 and inhibition of the NF-κB pathways. Respir. Res..

[44] Sauler M., Bazan I.S., Lee P.J. (2019). Cell Death in the Lung: The Apoptosis-Necroptosis Axis. Annu. Rev. Physiol..

[45] Feng H., Li M., Altawil A., Yin Y., Zheng R., Kang J. (2021). Cigarette smoke extracts induce apoptosis in Raw264.7 cells via endoplasmic reticulum stress and the intracellular Ca(2+)/P38/STAT1 pathway. Toxicol. Vitr..

[46] Luan G., Zhu Z., Wu K., Yin S. (2022). Theaflavin-3,3′-digallate attenuates cigarette smoke extract-induced pulmonary emphysema in mice by suppressing necroptosis. Exp. Ther. Med..

[47] Zhang M.Y., Jiang Y.X., Yang Y.C., Liu J.Y., Huo C., Ji X.L., Qu Y.Q. (2021). Cigarette smoke extract induces pyroptosis in human bronchial epithelial cells through the ROS/NLRP3/caspase-1 pathway. Life Sci..

[48] Yoshida M., Minagawa S., Araya J., Sakamoto T., Hara H., Tsubouchi K., Hosaka Y., Ichikawa A., Saito N., Kadota T. (2019). Involvement of cigarette smoke-induced epithelial cell ferroptosis in COPD pathogenesis. Nat. Commun..

[49] Bagam P., Kaur G., Singh D.P., Batra S. (2020). In vitro study of the role of FOXO transcription factors in regulating cigarette smoke extract-induced autophagy. Cell Biol. Toxicol..

[50] Vij N., Chandramani-Shivalingappa P., Van Westphal C., Hole R., Bodas M. (2018). Cigarette smoke-induced autophagy impairment accelerates lung aging, COPD-emphysema exacerbations and pathogenesis. Am. J. Physiol. Cell Physiol..

[51] Vu T., Jin L., Datta P.K. (2016). Effect of Cigarette Smoking on Epithelial to Mesenchymal Transition (EMT) in Lung Cancer. J. Clin. Med..

[52] Zuo H., Trombetta-Lima M., Heijink I.H., van der Veen C., Hesse L., Faber K.N., Poppinga W.J., Maarsingh H., Nikolaev V.O., Schmidt A.M. (2020). A-Kinase Anchoring Proteins Diminish TGF-β(1)/Cigarette Smoke-Induced Epithelial-To-Mesenchymal Transition. Cells.

[53] Carlier F.M., Dupasquier S., Ambroise J., Detry B., Lecocq M., Biétry-Claudet C., Boukala Y., Gala J.L., Bouzin C., Verleden S.E. (2020). Canonical WNT pathway is activated in the airway epithelium in chronic obstructive pulmonary disease. EBioMedicine.

[54] Alexandrov L.B., Ju Y.S., Haase K., Van Loo P., Martincorena I., Nik-Zainal S., Totoki Y., Fujimoto A., Nakagawa H., Shibata T. (2016). Mutational signatures associated with tobacco smoking in human cancer. Science.

[55] Cao C., Tian B., Geng X., Zhou H., Xu Z., Lai T., Wu Y., Bao Z., Chen Z., Li W. (2021). IL-17-Mediated Inflammation Promotes Cigarette Smoke-Induced Genomic Instability. Cells.

[56] Jiao Y., Liu C., Cui F.M., Xu J.Y., Tong J., Qi X.F., Wang L.L., Zhu W. (2015). Long intergenic non-coding RNA induced by X-ray irradiation regulates DNA damage response signaling in the human bronchial epithelial BEAS-2B cell line. Oncol. Lett..

[57] Gao S., Lin H., Yu W., Zhang F., Wang R., Yu H., Qian B. (2019). LncRNA LCPAT1 is involved in DNA damage induced by CSE. Biochem. Biophys. Res. Commun..

[58] Palamidas A., Tsikrika S., Katsaounou P.A., Vakali S., Gennimata S.A., Kaltsakas G., Gratziou C., Koulouris N. (2017). Acute effects of short term use of ecigarettes on Airways Physiology and Respiratory Symptoms in Smokers with and without Airways Obstructive Diseases and in Healthy non smokers. Tob. Prev. Cessat.

[59] McConnell R., Barrington-Trimis J.L., Wang K., Urman R., Hong H., Unger J., Samet J., Leventhal A., Berhane K. (2017). Electronic Cigarette Use and Respiratory Symptoms in Adolescents. Am. J. Respir. Crit. Care Med..

[60] Bowler R.P., Hansel N.N., Jacobson S., Graham B.R., Make B.J., Han M.K., O’Neal W.K., Oelsner E.C., Casaburi R., Barjaktarevic I. (2017). Electronic Cigarette Use in US Adults at Risk for or with COPD: Analysis from Two Observational Cohorts. J. Gen. Intern. Med..

[61] Garcia-Arcos I., Geraghty P., Baumlin N., Campos M., Dabo A.J., Jundi B., Cummins N., Eden E., Grosche A., Salathe M. (2016). Chronic electronic cigarette exposure in mice induces features of COPD in a nicotine-dependent manner. Thorax.

[62] Reinikovaite V., Rodriguez I.E., Karoor V., Rau A., Trinh B.B., Deleyiannis F.W., Taraseviciene-Stewart L. (2018). The effects of electronic cigarette vapour on the lung: Direct comparison to tobacco smoke. Eur. Respir. J..

[63] Olfert I.M., DeVallance E., Hoskinson H., Branyan K.W., Clayton S., Pitzer C.R., Sullivan D.P., Breit M.J., Wu Z., Klinkhachorn P. (2018). Chronic exposure to electronic cigarettes results in impaired cardiovascular function in mice. J. Appl. Physiol..

[64] Reid K.M., Forrest J.R., Porter L. (2018). Tobacco Product Use among Youths with and Without Lifetime Asthma—Florida, 2016. MMWR Morb. Mortal. Wkly. Rep..

[65] Bircan E., Bezirhan U., Porter A., Fagan P., Orloff M.S. (2021). Electronic cigarette use and its association with asthma, chronic obstructive pulmonary disease (COPD) and asthma-COPD overlap syndrome among never cigarette smokers. Tob. Induc. Dis..

[66] Lappas A.S., Tzortzi A.S., Konstantinidi E.M., Teloniatis S.I., Tzavara C.K., Gennimata S.A., Koulouris N.G., Behrakis P.K. (2018). Short-term respiratory effects of e-cigarettes in healthy individuals and smokers with asthma. Respirology.

[67] Polosa R., Morjaria J.B., Caponnetto P., Caruso M., Campagna D., Amaradio M.D., Ciampi G., Russo C., Fisichella A. (2016). Persisting long term benefits of smoking abstinence and reduction in asthmatic smokers who have switched to electronic cigarettes. Discov. Med..

[68] Iskandar A.R., Gonzalez-Suarez I., Majeed S., Marescotti D., Sewer A., Xiang Y., Leroy P., Guedj E., Mathis C., Schaller J.P. (2016). A framework for in vitro systems toxicology assessment of e-liquids. Toxicol. Mech. Methods.

[69] Anthérieu S., Garat A., Beauval N., Soyez M., Allorge D., Garçon G., Lo-Guidice J. (2017). Comparison of cellular and transcriptomic effects between electronic cigarette vapor and cigarette smoke in human bronchial epithelial cells. Toxicol. Vitr..

[70] Lerner C.A., Sundar I.K., Yao H., Gerloff J., Ossip D.J., McIntosh S., Robinson R., Rahman I. (2015). Vapors Produced by Electronic Cigarettes and E-Juices with Flavorings Induce Toxicity, Oxidative Stress, and Inflammatory Response in Lung Epithelial Cells and in Mouse Lung. PLoS ONE.

[71] Scott A., Lugg S.T., Aldridge K., Lewis K.E., Bowden A., Mahida R.Y., Grudzinska F.S., Dosanjh D., Parekh D., Foronjy R. (2018). Pro-inflammatory effects of e-cigarette vapour condensate on human alveolar macrophages. Thorax.

[72] O’Farrell H.E., Brown R., Brown Z., Milijevic B., Ristovski Z.D., Bowman R.V., Fong K.M., Vaughan A., Yang I.A. (2021). E-cigarettes induce toxicity comparable to tobacco cigarettes in airway epithelium from patients with COPD. Toxicol. Vitr..

[73] Larcombe A.N., Janka M.A., Mullins B.J., Berry L.J., Bredin A., Franklin P.J. (2017). The effects of electronic cigarette aerosol exposure on inflammation and lung function in mice. Am. J. Physiol.-Lung C.

[74] Glynos C., Bibli S., Katsaounou P., Pavlidou A., Magkou C., Karavana V., Topouzis S., Kalomenidis I., Zakynthinos S., Papapetropoulos A. (2018). Comparison of the effects of e-cigarette vapor with cigarette smoke on lung function and inflammation in mice. Am. J. Physiol.-Lung C.

[75] Crotty Alexander L.E., Drummond C.A., Hepokoski M., Mathew D., Moshensky A., Willeford A., Das S., Singh P., Yong Z., Lee J.H. (2018). Chronic inhalation of e-cigarette vapor containing nicotine disrupts airway barrier function and induces systemic inflammation and multiorgan fibrosis in mice. Am. J. Physiol. -Regul. Integr. Comp. Physiol..

[76] Lerner C.A., Rutagarama P., Ahmad T., Sundar I.K., Elder A., Rahman I. (2016). Electronic cigarette aerosols and copper nanoparticles induce mitochondrial stress and promote DNA fragmentation in lung fibroblasts. Biochem. Biophys. Res. Commun..

[77] Sun Y.W., Chen K.M., Atkins H., Aliaga C., Gordon T., Guttenplan J.B., El-Bayoumy K. (2021). Effects of E-Cigarette Aerosols with Varying Levels of Nicotine on Biomarkers of Oxidative Stress and Inflammation in Mice. Chem. Res. Toxicol..

[78] Yogeswaran S., Muthumalage T., Rahman I. (2021). Comparative Reactive Oxygen Species (ROS) Content among Various Flavored Disposable Vape Bars, including Cool (Iced) Flavored Bars. Toxics.

[79] Lamb T., Muthumalage T., Rahman I. (2020). Pod-based menthol and tobacco flavored e-cigarettes cause mitochondrial dysfunction in lung epithelial cells. Toxicol. Lett..

[80] Wavreil F.D.M., Heggland S.J. (2020). Cinnamon-flavored electronic cigarette liquids and aerosols induce oxidative stress in human osteoblast-like MG-63 cells. Toxicol. Rep..

[81] Muthumalage T., Prinz M., Ansah K.O., Gerloff J., Sundar I.K., Rahman I. (2017). Inflammatory and Oxidative Responses Induced by Exposure to Commonly Used e-Cigarette Flavoring Chemicals and Flavored e-Liquids without Nicotine. Front. Physiol..

[82] Ganapathy V., Manyanga J., Brame L., McGuire D., Sadhasivam B., Floyd E., Rubenstein D.A., Ramachandran I., Wagener T., Queimado L. (2017). Electronic cigarette aerosols suppress cellular antioxidant defenses and induce significant oxidative DNA damage. PLoS ONE.

[83] Anderson C., Majeste A., Hanus J., Wang S. (2016). E-Cigarette Aerosol Exposure Induces Reactive Oxygen Species, DNA Damage, and Cell Death in Vascular Endothelial Cells. Toxicol. Sci..

[84] Lee H.W., Park S.H., Weng M.W., Wang H.T., Huang W.C., Lepor H., Wu X.R., Chen L.C., Tang M.S. (2018). E-cigarette smoke damages DNA and reduces repair activity in mouse lung, heart, and bladder as well as in human lung and bladder cells. Proc. Natl. Acad. Sci. USA.

[85] Canistro D., Vivarelli F., Cirillo S., Babot Marquillas C., Buschini A., Lazzaretti M., Marchi L., Cardenia V., Rodriguez-Estrada M.T., Lodovici M. (2017). E-cigarettes induce toxicological effects that can raise the cancer risk. Sci. Rep..

[86] Alanazi H., Park H.J., Chakir J., Semlali A., Rouabhia M. (2018). Comparative study of the effects of cigarette smoke and electronic cigarettes on human gingival fibroblast proliferation, migration and apoptosis. Food Chem. Toxicol..

[87] Rouabhia M., Park H.J., Semlali A., Zakrzewski A., Chmielewski W., Chakir J. (2017). E-Cigarette Vapor Induces an Apoptotic Response in Human Gingival Epithelial Cells through the Caspase-3 Pathway. J. Cell. Physiol..

[88] Zahedi A., Phandthong R., Chaili A., Remark G., Talbot P. (2018). Epithelial-to-mesenchymal transition of A549 lung cancer cells exposed to electronic cigarettes. Lung Cancer.

[89] Park H.R., Vallarino J., O’Sullivan M., Wirth C., Panganiban R.A., Webb G., Shumyatcher M., Himes B.E., Park J.A., Christiani D.C. (2021). Electronic cigarette smoke reduces ribosomal protein gene expression to impair protein synthesis in primary human airway epithelial cells. Sci. Rep..

[90] Solleti S.K., Bhattacharya S., Ahmad A., Wang Q., Mereness J., Rangasamy T., Mariani T.J. (2017). MicroRNA expression profiling defines the impact of electronic cigarettes on human airway epithelial cells. Sci. Rep..

[91] Le H.H.T., Liu C.W., Denaro P.R., Jousma J., Shao N.Y., Rahman I., Lee W.H. (2021). Genome-wide differential expression profiling of lncRNAs and mRNAs in human induced pluripotent stem cell-derived endothelial cells exposed to e-cigarette extract. Stem Cell Res. Ther..

[92] Ferger B., Spratt C., Earl C.D., Teismann P., Oertel W.H., Kuschinsky K. (1998). Effects of nicotine on hydroxyl free radical formation in vitro and on MPTP-induced neurotoxicity in vivo. Naunyn Schmiedebergs Arch. Pharm..

[93] Vijg J. (2021). From DNA damage to mutations: All roads lead to aging. Ageing Res. Rev..

[94] Higham A., Rattray N.J.W., Dewhurst J.A., Trivedi D.K., Fowler S.J., Goodacre R., Singh D. (2016). Electronic cigarette exposure triggers neutrophil inflammatory responses. Respir. Res..

[95] Chen I., Todd I., Tighe P.J., Fairclough L.C. (2020). Electronic cigarette vapour moderately stimulates pro-inflammatory signalling pathways and interleukin-6 production by human monocyte-derived dendritic cells. Arch. Toxicol..

[96] Singh D.P., Begum R., Kaur G., Bagam P., Kambiranda D., Singh R., Batra S. (2021). E-cig vapor condensate alters proteome and lipid profiles of membrane rafts: Impact on inflammatory responses in A549 cells. Cell Biol. Toxicol..

[97] Hubner R.H., Schwartz J.D., De Bishnu P., Ferris B., Omberg L., Mezey J.G., Hackett N.R., Crystal R.G. (2009). Coordinate control of expression of Nrf2-modulated genes in the human small airway epithelium is highly responsive to cigarette smoking. Mol. Med..

[98] Marshall K., Liu Z., Olfert I.M., Gao W. (2020). Chronic electronic cigarette use elicits molecular changes related to pulmonary pathogenesis. Toxicol. Appl. Pharmacol..

[99] Kuntic M., Oelze M., Steven S., Kröller-Schön S., Stamm P., Kalinovic S., Frenis K., Vujacic-Mirski K., Bayo Jimenez M.T., Kvandova M. (2020). Short-term e-cigarette vapour exposure causes vascular oxidative stress and dysfunction: Evidence for a close connection to brain damage and a key role of the phagocytic NADPH oxidase (NOX-2). Eur. Heart J..

[100] Singh R.K., Kumar S., Gautam P.K., Tomar M.S., Verma P.K., Singh S.P., Kumar S., Acharya A. (2017). Protein kinase C-alpha and the regulation of diverse cell responses. Biomol. Concepts.

[101] Bootman M.D., Bultynck G. (2020). Fundamentals of Cellular Calcium Signaling: A Primer. Cold Spring Harb. Perspect. Biol..

[102] Ghosh A., Beyazcicek O., Davis E.S., Onyenwoke R.U., Tarran R. (2021). Cellular effects of nicotine salt-containing e-liquids. J. Appl. Toxicol..

[103] Ghosh A., Coakley R.D., Ghio A.J., Muhlebach M.S., Esther C.R., Alexis N.E., Tarran R. (2019). Chronic E-Cigarette Use Increases Neutrophil Elastase and Matrix Metalloprotease Levels in the Lung. Am. J. Respir. Crit. Care.

[104] Ung T.T., Nguyen T.T., Li S., Han J., Jung Y.D. (2021). Nicotine stimulates CYP1A1 expression in human hepatocellular carcinoma cells via AP-1, NF-κB, and AhR. Toxicol. Lett..

[105] Sun Y.W., Kosinska W., Guttenplan J.B. (2019). E-cigarette Aerosol Condensate Enhances Metabolism of Benzo(a)pyrene to Genotoxic Products, and Induces CYP1A1 and CYP1B1, Likely by Activation of the Aryl Hydrocarbon Receptor. Int. J. Environ. Res. Public Health.

[106] Kwon H.J., Oh Y.T., Park S., Kim S.S., Park J., Yin J., Hong J.H., Kim C.I., Ryu H., Park J.B. (2021). Analysis of electric cigarette liquid effect on mouse brain tumor growth through EGFR and ERK activation. PLoS ONE.

[107] Wang T.W., Neff L.J., Park-Lee E., Ren C., Cullen K.A., King B.A. (2020). E-cigarette Use among Middle and High School Students—United States, 2020. MMWR Morb. Mortal. Wkly. Rep..

[108] Chand H.S., Muthumalage T., Maziak W., Rahman I. (2020). Pulmonary Toxicity and the Pathophysiology of Electronic Cigarette, or Vaping Product, Use Associated Lung Injury. Front. Pharmacol..

